# The risk factors and predictive model for cardiac valve calcification in patients on maintenance peritoneal dialysis: a single-center retrospective study

**DOI:** 10.1080/0886022X.2023.2271069

**Published:** 2023-10-23

**Authors:** Yuxi Wang, Quanquan Shen, Junni Wang, Shilong Xiang, Yaomin Wang, Xiaohui Zhang, Jianghua Chen, Fei Han

**Affiliations:** a Kidney Disease Center, The First Affiliated Hospital, Zhejiang University School of Medicine; Institute of Nephrology, Zhejiang University; Key Laboratory of Kidney Disease Prevention and Control Technology, Zhejiang Province; Zhejiang Clinical Research Center of Kidney and Urinary System Disease, Hangzhou, Zhejiang, China; b Department of Nephrology, Zhejiang Provincial People’s Hospital, People’s Hospital of Hangzhou Medical College, Hangzhou, Zhejiang, China

**Keywords:** Cardiovascular calcification, peritoneal dialysis, nomogram model

## Abstract

**Background:**

Cardiovascular calcification includes cardiac valve calcification (CVC) and vascular calcification. We aimed to analyze risk factors for CVC, and construct a predictive model in maintenance peritoneal dialysis (MPD) patients.

**Methods:**

We retrospectively analyzed MPD patients who began peritoneal dialysis between January 2014 and September 2021. Patients were randomly assigned to the derivation cohort and validation cohort in a 7:3 ratio. The patients in the derivation cohort were divided into the CVC group and non-CVC group. Logistic regression was used to analyze risk factors, then the rms package in R language was used to construct a nomogram model to predict CVC.

**Results:**

1,035 MPD patients were included, with the age of 50.0 ± 14.2 years and 632 males (61.1%). Their median follow-up time was 25 (12, 46) months. The new-onset CVC occurred in 128 patients (12.4%). In the derivation cohort, multivariate logistic regression indicated old age, female, high systolic blood pressure (SBP), high calcium-phosphorus product (Ca × P), high Charlson comorbidity index (CCI) and long dialysis time were independent risk factors for CVC (*p* < 0.05). We constructed a nomogram model for predicting CVC in the derivation cohort, with a C index of 0.845 (95% CI 0.803–0.886). This model was validated with a C index of 0.845 (95%CI 0.781–0.909) in the validation cohort.

**Conclusion:**

We constructed a nomogram model for CVC in MPD patients, using independent risk factors including age, sex, SBP, Ca × P, CCI and dialysis time. This model achieved high efficiency in CVC prediction.

## Introduction

Cardiac valve calcification (CVC) is common in patients with chronic kidney disease (CKD) and has a significantly higher incidence than that in the general population [1,2]. CVC, the main reason of cardiovascular disease (CVD) and all-cause death in CKD patients [1], leads to cardiovascular events such as valve stenosis, arrhythmias, heart failure, and even sudden death. It is also a strong predictor for diseases such as atherosclerosis, coronary heart disease, and left ventricular hypertrophy which increase CVD incidence and mortality [3,4]. In dialysis populations, especially peritoneal dialysis (PD) patients, CVC is closely associated with increased risk of CVD and all-cause death [5–7]. Clinical guidelines recommend echocardiography in CKD patients to detect CVC, when CKD patients comorbid with CVC are considered at high risk of CVD [8,9].

Traditional risk factors for CVC include advanced age, diabetes, hypertension, hyperlipidemia, obesity, and smoking [10,11]. However, the incidence of CVC in CKD patients is 5-10 times higher than in patients without CKD [2], suggesting that traditional risk factors do not fully explain the causes. Cohort studies in CKD patients showed that after adjustment of traditional cardiovascular risk factors, renal function itself was closely related to the occurrence of CVC [12,13]. In addition, hyperphosphatemia is highly associated with the incidence of CVC. Adeney et al. suggested that after adjusting for demographics and kidney function in CKD stage 3 patients, the incidence of aortic valve calcification (AVC) and mitral valve calcification (MVC) increased by 25% and 62%, respectively when serum phosphate increased per 1mg/dl [14]. As for CKD patients who started dialysis, prolonged dialysis age is also an independent risk factor for CVC. The study by Tian et al. on 194 PD patients indicated that dialysis duration was longer in the new-onset CVC group compared with a non-CVC group [15]. A domestic study of risk factors associated with CVC in 110 adult hemodialysis (HD) patients showed that long-term dialysis was a major risk factor for CVC [16]. In this study, we retrospectively analyzed the risk factors for CVC in maintenance peritoneal dialysis (MPD) patients in our center. We further established a nomogram model for predicting new-onset CVC in MPD patients, so that it can provide a basis for the formulation of effective strategies for prevention and treatment of CVC in MPD patients.

## Materials and methods

### Study population

We retrospectively screened and enrolled MPD Patients who started peritoneal dialysis treatment from January 2014 to September 2021 at the Kidney Disease Center of the First Affiliated Hospital, Zhejiang University School of Medicine. The inclusion criteria were maintenance PD for more than 3 months, age over 18 years, and having echocardiography results after initiation of PD therapy with complete clinical key data. The exclusion criteria were as follows: 1) acute kidney injury with recovery of renal function after PD treatment; 2) valve calcification present before initiation of PD; 3) history of heart surgery.

We collected and recorded the following information of patients: 1) information on age, gender, weight, height, blood pressure, primary disease, history of hypertension, diabetes, etc. at the beginning of PD treatment; 2) fasting blood indicators prior to PD catheterization, including hemoglobin (Hb), albumin (Alb), blood glucose (BG), serum creatinine (sCr), uric acid (UA), potassium (K), calcium (Ca), phosphorus (P), alkaline phosphatase (ALP), triglyceride (TG), total cholesterol (TC), high-density lipoprotein cholesterol (HDL-c), low-density lipoprotein cholesterol (LDL-c), very low density lipoprotein cholesterol (VLDL-c), intact parathyroid hormone (iPTH), etc.; 3) estimated glomerular filtration rate (eGFR), body mass index (BMI), calcium-phosphate product (Ca × P), peritoneal Kt/V, renal Kt/V and total Kt/V, calculated according to the following formula: eGFR was estimated using the Chronic Kidney Disease Epidemiological Collaborative equation (CKD-EPI equation) [17]; body-mass index (BMI) (kg/m^2^)=body weight (kg)/height^2^ (m^2^); Ca × P (mg^2^/dl^2^)= Ca(mg/dl)×P(mg/dl); peritoneal Kt/V was calculated from pre-dialysis and post-dialysis urea concentration, renal Kt/V was calculated from urea clearance rate [18], total Kt/V = peritoneal Kt/V + renal Kt/V; 4) dialysis time, as for patients who developed CVC, the dialysis time was the time from PD start to the diagnosis of CVC; for patients who did not develop CVC, the dialysis time was the time from PD start to the end of observation.

Patients underwent echocardiography at the beginning of PD as baseline and when they accepted at least 3-month PD therapy. Echocardiography measurement parameters included interventricular septum end-diastolic thickness (IVSd), interventricular septum end-systolic thickness (IVSs), left ventricular end-diastolic dimension (LVEDD), left ventricular end-systolic dimension (LVESD), left ventricular posterior wall end-diastolic thickness (LVPWd), left ventricular posterior wall end-systolic thickness (LVPWs), left ventricular ejection fraction (LVEF), left ventricular fractional shortening (LVFS), left atrial diameter (LAD) and cardiac valve calcification (CVC). LVFS was calculated by (LVEDD-LVESD)/LVEDD. CVC was defined as the presence of one or more strong echoes larger than 1 mm on the aortic valve, mitral valve, or annulus of the heart.

For all included MPD patients, concomitant disease information on their Charlson comorbidity index (CCI) scale was collected and scored at the start of PD therapy [19]. CCI score includes 19 common medical concomitant conditions including: 1) score 1 point: myocardial infarct, congestive heart failure, peripheral vascular disease, cerebrovascular disease, dementia, chronic pulmonary disease, connective tissue disease, ulcer disease, mild liver disease, diabetes; 2) score 2 points: hemiplegia, moderate or severe renal disease, diabetes with end organ damage, any tumor, leukemia, lymphoma; 3) score 3 points: moderate or severe liver disease; 4) score 6 points: metastatic solid tumors, acquired immunodeficiency syndrome [19].

All data were statistically analyzed using SPSS 25.0 statistical software and R software (version 4.3). Measurements that followed a normal distribution were expressed in the form of mean ± SD. Non-normal distribution measurement data were expressed in the form of median (interquartile range). The comparison of measurement data that conformed to the normal distribution and variance between groups used the independent sample *t*-test, otherwise using Mann-Whitney *U* test; the count data were expressed as cases and percentages (%), and the χ^2^ test was used for comparison between the two groups.

To construct a nomogram model and examine the external validity of the model, our cohort was split into a derivation cohort and a validation cohort in a 7:3 ratio. The variables in the derivation cohort with *p* < 0.05 in univariate Logistic regression analysis were incorporated into a multivariate logistic regression equation to analyze the independent influencing factors for CVC in MPD patients. Finally, the rms package in R language was used to draw a nomogram model to predict CVC in MPD patients. To assess the discrimination and calibration of the nomogram model, we employed the ROCR package and the rms package in R language. Consequently, we obtained the area under the curve (AUC) for both cohorts, as well as the relationship between predicted probabilities and actual probabilities. A *p* < 0.05 was considered statistically significant.

## Results

**Figure 1.**
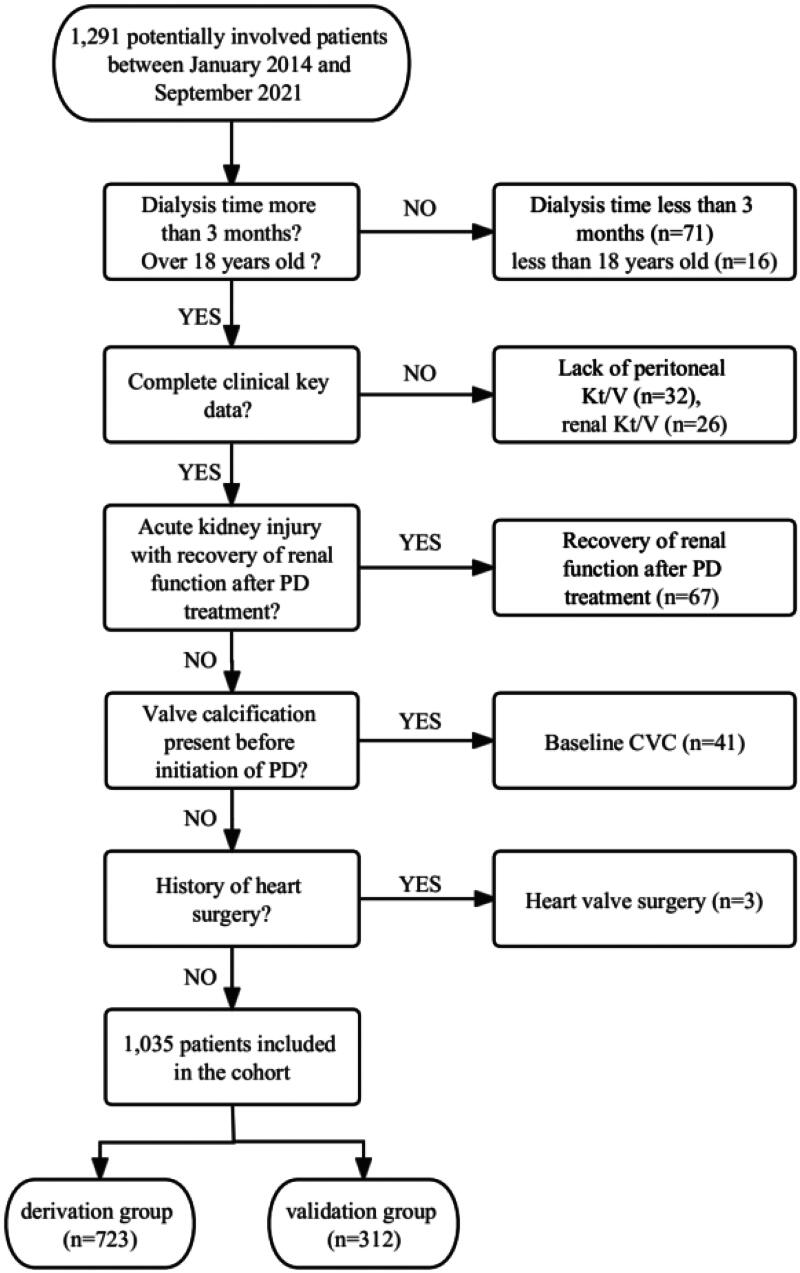
Flowchart of the recruitment.

We retrospectively screened 1,291 MPD patients, the recruitment flowchart is shown in Figure 1. A total of 1,035 MPD patients were finally included. Their average age was 50.0 ± 14.2 years, including 632 males (61.1%). Their median follow-up time was 25 (12, 46) months. There were 128 patients (12.4%) diagnosed with new-onset CVC during follow up, including 75 patients of mitral valve calcification (MVC), 40 patients of aortic valve calcification (AVC), and 13 patients of both mitral and aortic valves calcification. For the echocardiography parameters, MPD patients in CVC group had lower levels of cardiac structural indexes including IVSd (8.6mm vs 10.0mm, *p* < 0.001), and higher levels of IVSs (14.5mm vs 13.5mm, *p* < 0.001), LVPWd (10.0mm vs 9.5mm, *p* < 0.001), LVPWs (15.9mm vs 15.0mm, *p* = 0.01) and LAD (37.0mm vs 34.0mm, *p* < 0.001), shown in Table 1.

**Table 1.** The cardiac structural indexes in MPD patients with or without CVC.

Variables	Total cases(*n* = 1,035)	Without CVC(*n* = 907)	With CVC(*n* = 128)	*p*
IVSd (mm)	9.7 (8.4, 11.0)	10.0 (8.7, 11.0)	8.6 (6.8, 9.8)	< 0.001
IVSs (mm)	13.6 (12.0, 15.4)	13.5 (12.0, 15.3)	14.5 (13.0, 16.4)	< 0.001
LVEDD (mm)	49.0 (45.0, 53.1)	49.2 (45.0, 53.2)	49.0 (44.6, 52.8)	0.44
LVESD (mm)	31.0 (27.8, 34.3)	31.0 (28.0, 34.3)	30.6 (27.2, 34.2)	0.22
LVPWd (mm)	9.7 (8.7, 11.0)	9.5 (8.7, 10.9)	10.0 (9.1, 12.0)	< 0.001
LVPWs (mm)	15.0 (13.0, 17.0)	15.0 (13.0, 17.0)	15.9 (13.0, 17.9)	0.01
LVFS (%)	36.5 (33.3, 39.8)	36.4 (33.2, 39.7)	37.3 (33.4, 40.6)	0.17
LVEF (%)	66.0 (61.5, 70.0)	66.0 (61.5, 70.0)	67.5 (61.0, 71.0)	0.25
LAD (mm)	34.0 (30.0, 38.0)	33.7 (29.9, 37.0)	37.0 (33.0, 42.0)	< 0.001

MPD: maintenance peritoneal dialysis; CVC: cardiac valve calcification; IVSd: interventricular septum end-diastolic thickness; IVSs: interventricular septum end-systolic thickness; LVEDD: left ventricular end-diastolic dimension; LVESD: left ventricular end-systolic dimension; LVPWd: left ventricular posterior wall end-diastolic thickness; LVPWs: left ventricular posterior wall end-systolic thickness; LVFS: left ventricular fractional shortening; LVEF: left ventricular ejection fraction; LAD: left atrial diameter.

Patients were randomly assigned to the derivation cohort (*n* = 723) and the validation cohort (*n* = 312). There were no significant differences in clinical indicators between the derivation cohort and the validation cohort (shown in Table 2), suggesting two cohorts exhibited similar distributions of variables.

**Table 2.** Comparison of clinical indicators in derivation cohort and validation cohort.

Variables	derivation cohort	validation cohort	*P*
Case, *n* (%)	723 (69.9)	312 (30.1)	
Age, years	50 (40, 60)	51 (39, 61)	0.55
Female, *n* (%)	289 (40.0)	114 (36.5)	0.25
Diabetes, *n* (%)	92 (12.7)	36 (11.5)	0.60
Hypertension, *n* (%)	171 (23.7)	76 (24.4)	0.81
CVC, *n* (%)	88 (12.2)	40 (12.8)	0.77
SBP (mmHg)	140 (129, 155)	143 (130, 156)	0.66
DBP (mmHg)	86 (77, 96)	87 (77, 96)	0.73
BMI (kg/m^2^)	21.3 (19.4, 23.7)	21.9 (19.6, 23.7)	0.39
Hb (g/L)	83.5 (73.0, 96.0)	82.0 (71.0, 96.0)	0.23
Alb (g/L)	36.2 ± 5.7	36.5 ± 5.2	0.47
BG (mmol/L)	4.6 (4.2, 5.1)	4.6 (4.2, 5.1)	0.95
sCr (µmol/L)	710.0 (571.0, 909.5)	721.0 (585.0, 902.0)	0.59
eGFR (ml/min per 1.73 m^2^)	6.5 (5.0, 8.6)	6.3 (4.8, 8.7)	0.51
UA (µmol/L)	483.0 (402.3, 572.8)	478.0 (377.0, 573.0)	0.40
K (mmol/L)	4.5 (4.1, 4.9)	4.5 (4.0, 5.0)	0.91
Ca (mmol/L)	2.1 (2.0, 2.2)	2.1 (1.9, 2.2)	0.42
P (mmol/L)	1.7 (1.5, 2.1)	1.7 (1.4, 2.1)	0.86
Ca × P (mg^2^/dl^2^)	44.3 (37.1, 52.8)	44.5 (36.7, 52.1)	0.88
iPTH (pg/ml)	262.6 (136.0, 433.6)	239.0 (137.0, 412.0)	0.33
ALP (mmol/L)	68.0 (54.3, 85.8)	69.0 (55.5, 86.0)	0.69
TG (mmol/L)	1.3 (0.9, 1.8)	1.4 (1.0, 1.8)	0.40
TC (mmol/L)	4.0 (3.3, 4.7)	4.1 (3.4, 4.8)	0.37
HDL-c (mmol/L)	1.0 (0.9, 1.2)	1.0 (0.8, 1.3)	0.99
LDL-c (mmol/L)	2.2 (1.7, 2.8)	2.3 (1.7, 2.8)	0.35
VLDL-c (mmol/L)	0.7 (0.5, 0.9)	0.7 (0.5, 0.9)	0.36
CCI (points)	3 (2, 4)	3 (2, 4)	0.38
Dialysis time (months)	25.0 (12.8, 46.0)	25.0 (11.4, 45.8)	0.66
Peritoneal Kt/V	1.0 (0.8, 1.2)	0.9 (0.7, 1.2)	0.05
Renal Kt/V	0.9 (0.6, 1.3)	0.9 (0.6, 1.3)	0.81
Total Kt/V	2.0 (1.7, 2.3)	1.9 (1.6, 2.3)	0.22

SBP: systolic blood pressure; DBP: diastolic blood pressure; BMI: body mass index; Hb, hemoglobin; Alb: albumin; BG, blood glucose; sCr: serum creatinine; eGFR: estimated glomerular filtration rate; UA: uric acid; K: potassium; Ca: calcium; P: phosphorus; Ca × P: calcium-phosphorus product; iPTH: intact parathyroid hormone; ALP: alkaline phosphatase; TG, triglyceride; TC: total cholesterol; HDL-c: high density lipoprotein cholesterol; LDL-c: low density lipoprotein cholesterol; VLDL-c: very low density lipoprotein cholesterol; CCI: Charlson comorbidity index.

In derivation cohort, there were 88 patients had new-onset CVC during follow-up. The comparisons between patients with or without CVC are shown in Table 3. Compared with the non-CVC group, MPD patients in the CVC group were older (58 years vs 49 years, *p* < 0.001), and had a higher female ratio (55.7% vs 37.8%, *p* = 0.001), as well as higher levels of systolic blood pressure (SBP) (148mmHg vs 140mmHg, *p* = 0.01), Ca × P (47.0mg^2^/dl^2^ vs 43.7mg^2^/dl^2^, *p* = 0.02), CCI (4 points vs 2 points, *p* < 0.001), dialysis time (51.6 months vs 22.0 months, *p* < 0.001) and peritoneal Kt/V (1.1 vs 1.0, *p* = 0.01), but lower level of renal Kt/V (0.8 vs 0.9, *p* = 0.002).

**Table 3.** Comparison of clinical indicators in patients with or without CVC in derivation cohort.

Variables	Non-CVC	CVC	*P*
Case, *n* (%)	635 (87.8)	88 (12.2)	
Age, years	49 (39, 58)	58 (45, 68)	< 0.001
Female, *n* (%)	240 (37.8)	49 (55.7)	0.001
Diabetes, *n* (%)	79 (12.4)	13 (14.8)	0.54
Hypertension, *n* (%)	152 (23.9)	19 (21.6)	0.63
SBP (mmHg)	140 (128, 154)	148 (131, 162)	0.01
DBP (mmHg)	86 (77, 96)	84 (76, 95)	0.39
BMI (kg/m^2^)	21.3 (19.3, 23.6)	21.8 (20.1, 24.1)	0.39
Hb (g/L)	84 (73, 96)	83 (70, 95)	0.43
Alb (g/L)	36.2 ± 5.8	36.5 ± 5.1	0.64
BG (mmol/L)	4.6 (4.2, 5.1)	4.6 (4.2, 5.2)	0.97
sCr (µmol/L)	710.0 (573.5, 903.0)	712.5 (567.0, 967.0)	0.91
eGFR (ml/min per 1.73 m^2^)	6.6 (5.1, 8.6)	5.9 (4.6, 8.5)	0.12
UA (µmol/L)	485.5 (405.0, 574.8)	468.0 (371.0, 564.3)	0.07
K (mmol/L)	4.5 (4.1, 4.9)	4.6 (4.1, 5.0)	0.72
Ca (mmol/L)	2.1 (2.0, 2.2)	2.1 (2.0, 2.3)	0.11
P (mmol/L)	1.7 (1.4, 2.1)	1.8 (1.6, 2.1)	0.10
Ca × P (mg^2^/dl^2^)	43.7 (36.7, 52.6)	47.0 (41.0, 55.2)	0.02
iPTH (pg/ml)	260.5 (137.8, 431.5)	287.0 (113.0, 444.5)	0.998
ALP (mmol/L)	68.0 (54.0, 85.5)	68.0 (56.0, 86.0)	0.78
TG (mmol/L)	1.3 (0.9, 1.8)	1.3 (0.9, 1.7)	0.29
TC (mmol/L)	4.0 (3.3, 4.7)	4.3 (3.5, 4.8)	0.11
HDL-c (mmol/L)	1.0 (0.9, 1.2)	1.1 (0.9, 1.4)	0.06
LDL-c (mmol/L)	2.2 (1.7, 2.7)	2.4 (1.7, 2.9)	0.21
VLDL-c (mmol/L)	0.7 (0.5, 1.0)	0.7 (0.4, 0.9)	0.85
CCI (points)	2 (2, 4)	4 (2, 5)	< 0.001
Dialysis time (months)	22.0 (12.2, 43.0)	51.6 (26.0, 86.3)	< 0.001
Peritoneal Kt/V	1.0 (0.7, 1.2)	1.1 (0.8, 1.3)	0.01
Renal Kt/V	0.9 (0.7, 1.3)	0.8 (0.4, 1.1)	0.002
Total Kt/V	2.0 (1.7, 2.3)	1.9 (1.6, 2.2)	0.08

CVC,: cardiac valve calcification; SBP: systolic blood pressure; DBP: diastolic blood pressure; BMI: body mass index; Hb: hemoglobin; Alb: albumin; BG: blood glucose; sCr: serum creatinine; eGFR: estimated glomerular filtration rate; UA: uric acid; K, potassium; Ca: calcium; P: phosphorus; Ca × P: calcium-phosphorus product; iPTH: intact parathyroid hormone; ALP: alkaline phosphatase; TG: triglyceride; TC:total cholesterol; HDL-c: high density lipoprotein cholesterol; LDL-c: low density lipoprotein cholesterol; VLDL-c: very low density lipoprotein cholesterol; CCI: Charlson comorbidity index.

In the derivation cohort, CVC was used as the dependent variable for univariate logistic regression analysis, the variables with *p* < 0.05 included age (*p* < 0.001), female (*p* = 0.002), SBP (*p* = 0.02), UA (*p* = 0.03), Ca × P (*p* = 0.04), ALP (*p* = 0.04), HDL-c (*p* = 0.03), CCI (*p* < 0.001), dialysis time (*p* < 0.001), peritoneal Kt/V (*p* = 0.02) and renal Kt/V (*p* = 0.002). These variables were selected as covariates to enter the multivariate logistic regression analysis, the results showed that old age (OR = 1.04, 95%CI 1.01–1.06, *p* = 0.004), female (OR = 2.15, 95%CI 1.19–3.92, *p* = 0.01), high SBP (OR = 1.01, 95%CI 1.00–1.03, *p* = 0.048), high Ca × P (OR = 1.03, 95%CI 1.00–1.05, *p* = 0.02), high CCI (OR = 1.37, 95%CI 1.17–1.59, *p* < 0.001) and long dialysis time (OR = 1.04, 95%CI 1.03-1.05, *p* < 0.001) were independent risk factors for CVC in MPD patients, shown in Table 4.

**Table 4.** Influencing factors of CVC in derivation cohort (logistic regression equation, *n* = 723).

Variable	Univariate			multivariate		
	OR	95%CI	*P*	OR	95%CI	*P*
Age, years	1.04	1.02-1.06	<0.001	1.04	1.01-1.06	0.004
Female	2.07	1.32-3.24	0.002	2.15	1.19-3.92	0.01
Diabetes	1.22	0.65-2.30	0.54			
Hypertension	0.88	0.51-1.50	0.63			
SBP (mmHg)	1.01	1.00-1.02	0.02	1.01	1.00-1.03	0.048
DBP (mmHg)	0.998	0.98-1.01	0.77			
BMI (kg/m^2^)	1.02	0.95-1.09	0.59			
Hb (g/L)	0.99	0.98-1.01	0.37			
Alb (g/L)	1.01	0.97-1.05	0.64			
BG (mmol/L)	0.89	0.73-1.10	0.28			
sCr (µmol/L)	1.00	0.999-1.001	0.72			
eGFR (ml/min per 1.73 m^2^)	0.95	0.88-1.03	0.22			
UA (µmol/L)	0.998	0.996-1.00	0.03	0.998	0.996-1.001	0.16
K (mmol/L)	1.05	0.76-1.45	0.77			
Ca (mmol/L)	2.28	0.90-5.81	0.08			
P (mmol/L)	1.34	0.87-2.09	0.19			
Ca × P (mg^2^/dl^2^)	1.02	1.00-1.04	0.04	1.03	1.00-1.05	0.02
iPTH (pg/ml)	1.00	1.00-1.001	0.37			
ALP (mmol/L)	1.003	1.00-1.006	0.04	1.001	0.998-1.005	0.42
TG (mmol/L)	0.77	0.55-1.06	0.11			
TC (mmol/L)	1.02	0.91-1.13	0.77			
HDL-c (mmol/L)	2.01	1.08-3.74	0.03	1.17	0.56-2.44	0.67
LDL-c (mmol/L)	1.06	0.83-1.35	0.66			
VLDL-c (mmol/L)	0.89	0.52-1.53	0.68			
CCI (points)	1.21	1.10-1.34	<0.001	1.37	1.17-1.59	<0.001
Dialysis time (months)	1.04	1.03-1.05	<0.001	1.04	1.03-1.05	<0.001
Peritoneal Kt/V	1.96	1.10-3.51	0.02	0.84	0.36-1.95	0.68
Renal Kt/V	0.46	0.28-0.75	0.002	0.57	0.30-1.05	0.07
Total Kt/V	0.66	0.41-1.07	0.09			

CVC: cardiac valve calcification; SBP: systolic blood pressure; DBP: diastolic blood pressure; BMI: body mass index; Hb: hemoglobin; Alb: albumin; BG: blood glucose; sCr: serum creatinine; eGFR: estimated glomerular filtration rate; UA: uric acid; K: potassium; Ca: calcium; P: phosphorus; Ca × P: calcium-phosphorus product; iPTH: intact parathyroid hormone; ALP: alkaline phosphatase; TG: triglyceride; TC: total cholesterol; HDL-c: high density lipoprotein cholesterol; LDL-c: low density lipoprotein cholesterol; VLDL-c: very low density lipoprotein cholesterol; CCI:Charlson comorbidity index.

**Figure 2.**
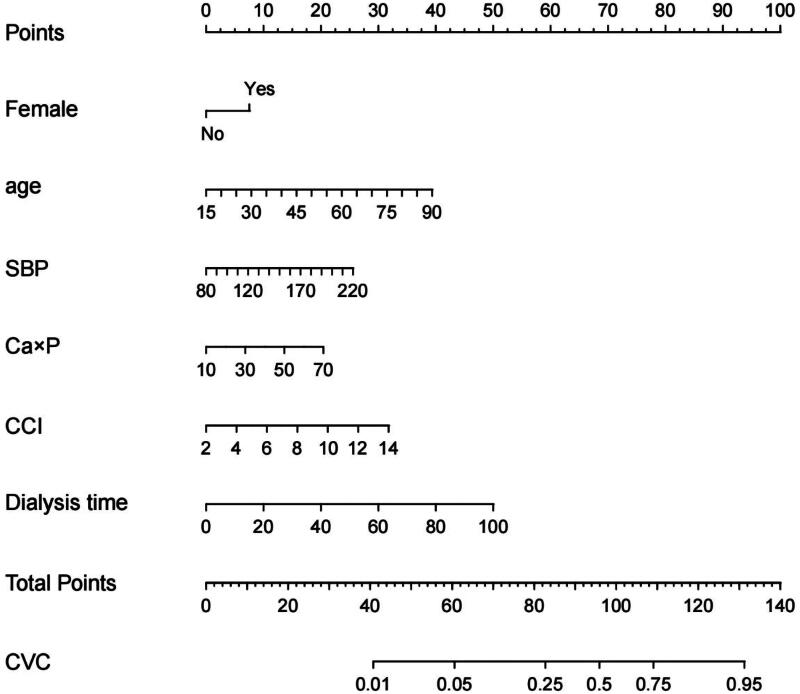
The constructed nomogram model for predicting new-onset CVC in MPD patients.

**Figure 3.**
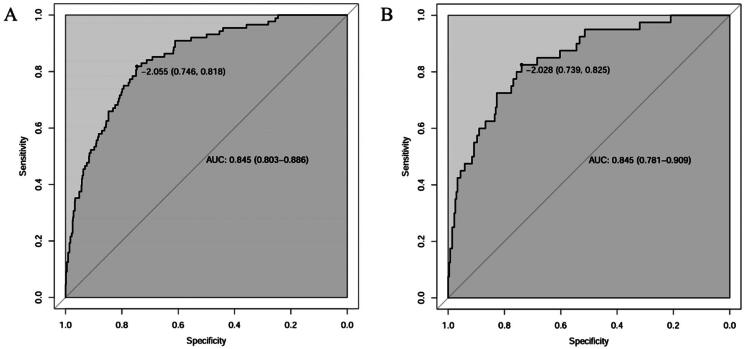
The discrimination of the constructed nomogram model. (A) ROC curves of the derivation cohort. The best cutoff value was -2.055, the related specificity was 0.746, the sensitivity was 0.818. (B) ROC curves of the validation cohort. The best cutoff value was -2.028, the related specificity was 0.739, the sensitivity was 0.825.

**Figure 4.**
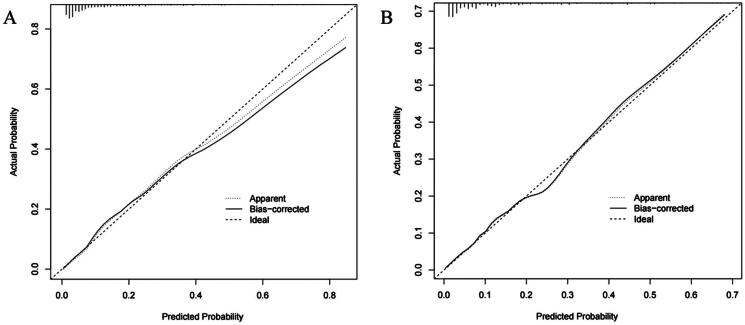
The validation of the constructed nomogram model. (A) Calibration curve of the derivation cohort. The χ^2^ test compared the difference between the predicted probability and the actual probability, *p* = 0.805. (B) Calibration curve of the validation cohort. *p* = 0.991.

According to the above logistic regression analysis results of the derivation cohort, a nomogram model was constructed to predict the new onset of CVC in MPD patients by rms package in R program. The results of the nomogram model showed that for each 5-year increase in age, the score increased by 2.5 points, also increased by 8 points for female patients; for every 10mmHg increase in SBP, the score increased 2 points; for every 10mg^2^/dl^2^ increase in Ca × P, the score increased by 3 points; for each point increase in CCI, the score increased by 3 points; for every 10 months increase in dialysis time, the nomogram model score increased by 5 points, shown in Figure 2. The C indices of the constructed nomogram model in the derivation cohort and the validation cohort were 0.845 (95% CI 0.803–0.886) and 0.845 (95%CI 0.781–0.909) respectively, indicating that the nomogram model is well differentiated, the best cutoff values of the nomogram were −2.055 and −2.028 (Figure 3A and B). Among the included patients in the two cohorts, the calibration curves used to predict the occurrence of CVC in MPD patients were straight lines with the slope close to 1 (Figure 4A and B), showing that the model had a good agreement between the predicted risk and the actual occurrence risk.

## Discussion

In this study, we found that after 25 (12, 46) months of follow-up, the incidence of new-onset CVC in MPD patients was 12.4%, of which MVC was the main type, accounting for 58.6%, AVC accounting for 31.3%, and both mitral and aortic valve calcification accounting for 10.1%. The factors including old age, female, high SBP, high Ca × P, high CCI and long dialysis time were independent risk factors for CVC in MPD patients.

CVD is the leading cause of death in MPD patients, and dialysis patients have a 10- to 20-fold greater risk of death from CVD than the general population [20,21]. CVC, including AVC and MVC, which causes structural changes in the heart, plays an important role in the development of CVD and is common in patients undergoing dialysis. In a cohort study of hemodialysis patients followed for 10 years in Japan, the incidence of CVC was as high as 57.5% [22]. Also, in the study of 194 MPD patients in China, after a follow-up of average 20.9 months, CVC was found in 50.0% of patients, of which AVC proportion was 76.29%, MVC was 10.51%, and 13.40% of patients had double-valve calcification, but this group of people was older than those in this study, their average age was 60.5 ± 13.0 years, and 62 patients (32%) had new-onset CVC after removing the peritoneal dialysis tube [15]. These two studies showed higher incidences of CVC than the incidence in our cohort, which may be related to the patients’ age and follow-up duration.

For echocardiography parameters, left ventricular hypertrophy was reported closely associated with CVC in CKD patients newly started dialysis [23]. This is also confirmed by the present study, which showed that the thickness of the left ventricular posterior wall in the CVC group was higher than that in the non-CVC group, both in the end-diastolic and end-systolic phases. Clinically, left atrial enlargement and left ventricular hypertrophy often accompany each other, and the increase in cardiac preload in CVC patients can cause increased pressure in the left atrium and left ventricle, and long-term high blood pressure leads to the increased left atrium and left ventricular volume and cardiac enlargement.

In a study of 135 CKD patients, old age was an independent factor for the occurrence of CVC [24]. In a retrospective study of dialysis patients in China, it was also found that among dialysis patients, the age of patients in the CVC group was 56.81 ± 11.78 years, which was significantly higher than the age of patients in the non-CVC group 49.74 ± 13.87 years [6]. Consistent with previous studies, this study confirmed that old age was an independent risk factor for CVC, which might be due to degenerative cardiovascular changes [25], and the increase in parathyroid hormone, causing calcium ions to migrate from bone to soft tissue, resulting in ectopic deposition of bone calcium. It might also be that the aging heart was in a state of hypoxia and acidosis, which was easy to cause the rupture of cardiac collagen fibers, and the formed gap combined with calcium salts, leading to the formation of CVC [26].

In the present study, we found that female was an independent risk factor for CVC in MPD patients. In a study of atherosclerotic disease risk factors, MVC and AVC were present in 67% and 63% of female patients, significantly higher than in male patients [27]. In studies by Tenenbaum A et al., Kanjanauthai S et al. and Adler Y et al., MVC was similarly found to be more common in women [28–30]. However, in the study of Owens et al., AVC was found to occur mostly in male patients [25]. It is generally believed that the effects of osteoporosis on women may make them particularly susceptible to calcifications within the heart valves, and other underlying mechanisms include gender-based differences in hormone levels and calcium and vitamin D supplementation [25]. In this study, we compared independent risk factors for CVC in male and female patients in Table 5, and found that female patients had a higher tendency of calcium-phosphorus product (*p* = 0.07) and longer duration of dialysis (*p* = 0.001). Also, females may have a higher risk of heart disease after menopause, and in our study, the median age of female participants was 50 (39, 60) years. Based on these reasons, the female gender may be a significant risk factors for CVC in this study.

**Table 5.** Comparison of independent risk factors between male and female patients.

Variables	male (*n* = 632)	female (*n* = 403)	*P*
Age, years	50 (40, 60)	50 (39,60)	0.49
SBP (mmHg)	141 (130, 155)	140 (127, 155)	0.37
Ca × P (mg^2^/dl^2^)	43.5 (36.7, 52.4)	45.7 (37.6, 53.1)	0.07
CCI (points)	3 (2, 4)	3 (2, 4)	0.02
Dialysis time (months)	22.6 (11.9, 42.0)	29.6 (13.2, 52.6)	0.001

SBP: systolic blood pressure; Ca × P: calcium-phosphorus product; CCI: Charlson comorbidity index.

In our study, high levels of SBP and Ca × P were also independent risk factors for CVC. SBP is an essential indicator of cardiovascular health. In a cross-sectional study of Japanese populations, it reported that SBP was more closely related to CVC than DBP [31]. Kimi S et al. also proved that the time between left ventricular and aortic systolic pressure peaks was associated with AVC [32]. Mineral metabolism disorder is also important in CVC for CKD patients. Studies have shown that blood calcium and phosphorus play an important role in the development of CVC in CKD patients [33,34]. Adeney et al. showed in 439 multi-ethnic CKD stage 3 patients without clinical CVD that even if blood phosphorus level was within normal range, the risk of AVC and MVC could increase by 25% and 62% respectively for every 1mg/dl increase in blood phosphorus [14]. In a time-dependent survival analysis, elevated blood phosphorus and Ca × P concentrations increased the risk of all-cause mortality in dialysis patients [35]. Wang et al. found in a study of 137 MPD patients that blood calcium, phosphorus, Ca × P, and PTH in CVC group were significantly higher than those in non-CVC group, and Ca × P was an independent risk factor for CVC in MPD patients [36].

The main limitation of this study is its single-center retrospective design. Second, due to limited follow-up time, we might miss more CVC cases that may develop in prolonged follow up. Third, medications like calcium-containing preparations and calcitriol may be related to CVC onset. However, due to the retrospective nature of this study, we could not collect the complete medication history, especially the regulations of these medications during follow-up.

In conclusion, we constructed a nomogram model for CVC in MPD patients, using independent risk factors including age, sex, SBP, Ca × P, CCI and dialysis time. This model achieved high efficiency in prediction with good discrimination and accuracy.
